# Mambalgin-3 potentiates human acid-sensing ion channel 1b under mild to moderate acidosis: Implications as an analgesic lead

**DOI:** 10.1073/pnas.2021581118

**Published:** 2021-02-19

**Authors:** Ben Cristofori-Armstrong, Elena Budusan, Lachlan D. Rash

**Affiliations:** ^a^School of Biomedical Sciences, Faculty of Medicine, The University of Queensland, St Lucia, QLD 4072, Australia

**Keywords:** acid-sensing ion channel, mambalgin, analgesic

## Abstract

Acid-sensing ion channels (ASICs) are expressed in the nervous system, activated by acidosis, and implicated in pain pathways. Mambalgins are peptide inhibitors of ASIC1 and analgesic in rodents via inhibition of centrally expressed ASIC1a and peripheral ASIC1b. This activity has generated interest in mambalgins as potential therapeutics. However, most mechanism and structure–activity relationship work on mambalgins has focused on ASIC1a, and neglected the peripheral analgesic target ASIC1b. Here, we compare mambalgin potency and mechanism of action at heterologously expressed rat and human ASIC1 variants. Unlike the nanomolar inhibition at ASIC1a and rodent ASIC1b, we find mambalgin-3 only weakly inhibits human ASIC1b and ASIC1b/3 under severe acidosis, but potentiates currents under mild/moderate acidosis. Our data highlight the importance of understanding the activity of potential ASIC-targeting pharmaceuticals at human channels.

Acid-sensing ion channels (ASICs) are homotrimers and heterotrimers activated by extracellular protonation during neurotransmission, ischemia, and inflammation and mediate excitatory sodium influx in pain signaling (1, 2). ASIC1a and ASIC1b are splice isoforms (differing in the N-terminal third) with different biophysical properties and expression profiles, that both respond to pathological acidosis (pH 6 to 7) (3–5). Isolated from mamba snake venoms, the mambalgin peptides inhibit ASIC1a (concentration that inhibits response by 50% [IC_50_]: rat, 3 to 55 nM, and human, 127 nM) and ASIC1b (IC_50_: rat, 38 to 192 nM) (6, 7). This unique pharmacology, together with genetic methods, showed that ASIC1b, along with ASIC3 (2), is involved in peripheral pain sensing in mice (mouse and rat ASIC1b are 99% identical with all six substitutions in the intracellular N terminus), and confirmed that ASIC1a contributes to central pain processing. Further work has corroborated the ASIC1b-mediated peripheral analgesia of mambalgins in animal models of acidosis-associated inflammation and pain, raising interest in their therapeutic potential (7–10). Mambalgins inhibit ASIC1a by decreasing the channel’s affinity for protons and stabilizing the resting state, thus acid shifting the pH dependence of activation (6, 11). This mechanism of inhibition has been extrapolated to ASIC1b. However, mambalgin pharmacology at the peripheral analgesic target ASIC1b has been somewhat neglected, and the effects of mambalgins on hASIC1b-containing channels have not been reported.

## Results and Discussion

**Fig. 1.**
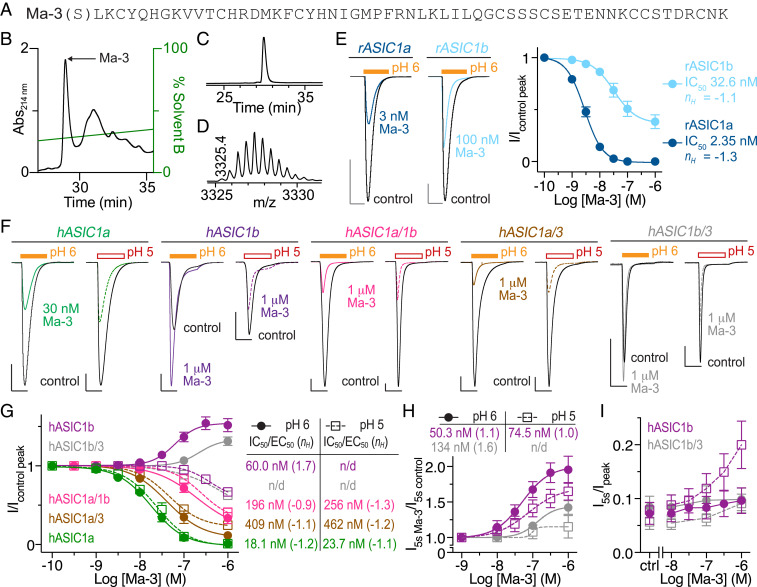
Production of Ma-3 and its species and subtype selectivity. (*A*) Recombinant Ma-3 has a vestigial N-terminal serine from protease cleavage. (*B*) Reversed-phase high-performance liquid chromatography (HPLC) chromatogram of crude cleavage reaction after affinity purification. (*C*) HPLC chromatogram showing >95% homogeneity and (*D*) matrix-assisted laser desorption ionization time-of-flight mass spectrometry spectrum showing the M+2H^+^ ion of recombinant Ma-3 (monoisotopic masses; observed, 6,648.8, and theoretical, 6,649.0). (*E*) Exemplar traces and concentration−response curves of Ma-3 at rat ASIC1a and ASIC1b. (*F*) Exemplar traces and (*G*) concentration−response curves of Ma-3 at human ASICs. Heteromers are from coinjection of two subtype messenger RNAs into a single oocyte. (*H* and *I*) Quantification of sustained current after 5 s of low-pH stimulus. Data are from 55-s Ma-3 application at pH 7.45, and channels were stimulated with pH 6 or 5 as indicated. (Scale bar: [*E* and *H*] abscissa 3 s, ordinate 600 nA.) For *E*, *G*, and *H*, Hill equation fits to the data with sufficient concentration ranges provide the Hill coefficient (*n*_*H*_) and IC_50_/EC_50 _(EC_50_, concentration resuling in 50% maximal effect).

To screen the activity of mambalgin-3 (Ma-3) across rat (r) and human (h) ASICs, we produced recombinant Ma-3 in *Escherichia coli* (yield: ∼200 μg/L; Fig. 1 *A*–*D*). Ma-3 inhibition of peak rASIC1a and rASIC1b currents expressed in *Xenopus* oocytes is comparable to native and synthetic mambalgins when applied at pH 7.45 and channels stimulated with pH 6 (Fig. 1*E*) (6, 7). Inhibition was also observed for hASIC1a, hASIC1a/1b, and hASIC1a/3 when stimulated with pH 5 or 6 with differing degrees of potency (Fig. 1 *F* and *G*). Surprisingly, Ma-3 activity at hASIC1b is opposite to that of ASIC1a and rASIC1b, with pH 7.45-applied Ma-3 potentiating pH 6 currents (Fig. 1 *F* and *G*). In contrast, with a more intense activation stimulus of pH 5, Ma-3 weakly inhibits hASIC1b. This activity profile is similar in hASIC1b/3 heteromers, albeit with weaker potency. Ma-3 also increased the steady-state current amplitude (i.e., end of the 5-s low-pH stimulus) for hASIC1b and hASIC1b/3 (Fig. 1 *F*, *H*, and *I*). Mambalgins have previously been reported to potentiate the peak current of rASIC2a:1a (thumb domain) chimeras and chicken ASIC1a point mutants (12, 13), but not wild-type human channels that may have physiological implications.

**Fig. 2.**
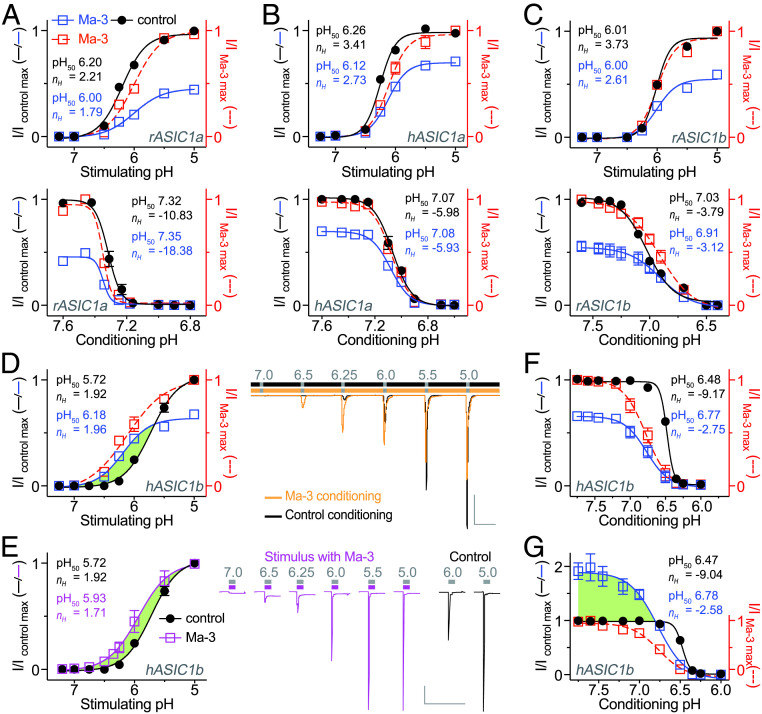
Channel-specific Ma-3 mechanism of action explains human ASIC1b potentiation. (*A*−*C*) The pH dependence of activation (*Upper*, conditioning pH 7.45) and SSD (*Lower*, stimulating pH 6) for (*A*) rat ASIC1a, (*B*) human ASIC1a, and (*C*) rat ASIC1b. (*D* and *E*) The pH dependence of activation with exemplar traces for human ASIC1b when Ma-3 is applied in (*D*) only the pH 7.45 conditioning solution and (*E*) each of the varied low pH stimulus solutions. (Gray scale bar: abscissa 30 s, ordinate 500 nA.) (*F* and *G*) The pH dependence of SSD when Ma-3 is applied in the pH 7.45 conditioning solution and channels stimulated with (*F*) pH 5 and (*G*) pH 6. The pH dependence curves are fit to current (I) normalized to either the maximal control current (left *y* axis of I/I_control_ _max_; solid lines) or the maximal current observed in the presence of Ma-3 (right *y* axis of I/I_Ma-3_ _max_; red dashed line). Where Ma-3 is present, concentrations are 3, 10, 30, and 1,000 nM for rASIC1a, hASIC1a, rASIC1b, and hASIC1b, respectively. Green shading in *D*, *E*, and *G* highlights pH conditions where Ma-3 potentiates hASIC1b currents. Hill equation fits to the data provide the color-matched pH_50_ and Hill coefficient (*n*_*H*_) for data normalized to maximal control currents.

We next explored the effect of Ma-3 on pH-dependent gating of rat and human ASIC1 homomers to understand the mechanism underlying hASIC1b potentiation. We first confirmed that subsaturating concentrations of Ma-3 shifted the pH_50_ (pH resulting in 50% maximal activation) of activation to more acidic values for both rASIC1a (from 95% CI 6.16–6.24 to 95% CI 5.91–6.08 in Ma-3) and hASIC1a (from 95% CI 6.23–6.28 to 95% CI 5.94–6.16 in Ma-3). Ma-3 had minimal effect on the pH_50_ of steady-state desensitization (SSD) for rASIC1a (from 95% CI 7.30–7.33 to 95% CI 7.34–7.36 in Ma-3) and hASIC1a (from 95% CI 7.05–7.08 to 95% CI 7.06–7.10 in Ma-3) (Fig. 2 *A* and *B*). We next examined the Ma-3 mechanism of action at rat and human ASIC1b where it has previously not been tested. In contrast to ASIC1a, Ma-3 did not shift the pH dependence of activation of rASIC1b (from 95% CI 5.99–6.03 to 95% CI 5.94–6.07 in Ma-3), and inhibited the current regardless of the pH used for stimulation (Fig. 2*C*). Furthermore, Ma-3 produced a modest acidic shift in the SSD properties of rASIC1b (from 95% CI 7.00–7.05 to 95% CI 6.82–6.99 in Ma-3) (Fig. 2*C*). The effect of Ma-3 on the pH-dependent properties of hASIC1b is strikingly different (Fig. 2 *D*–*G*) and explains our stimulation pH-dependent concentration−response data. We noted a significant alkaline shift (0.46 pH units) in the pH_50_ of activation (less protonation required to induce the same level of activation) (from 95% CI 5.67–5.78 to 95% CI 6.13–6.24 in Ma-3), combined with inhibition compared to control when stimulated with pH below ∼5.75 (Fig. 2*D*). We then examined whether Ma-3 also has an effect when present only in the low-pH stimulating solution. Coapplication of Ma-3 with low pH resulted in a 0.21-unit alkaline shift of pH activation (from 95% CI 5.67–5.78 to 95% CI 5.80–6.06 in Ma-3) (Fig. 2*E*). However, in contrast to Ma-3 application in the conditioning solution, we did not see inhibition of current at lower pH stimuli. The presence of Ma-3 alkaline shifted the pH dependence of SSD of hASIC1b when channels are stimulated with pH 5 (Fig. 2*F*) (from 95% CI 6.47–6.49 to 95% CI 6.70–6.83 in Ma-3) yet resulted in current inhibition, possibly stabilizing the desensitized state similar to the effect of PcTx1 on ASIC1a (14). By contrast, the alkaline shift in SSD of hASIC1b when stimulated with pH 6 (Fig. 2*G*) (from 95% CI 6.46–6.48 to 95% CI 6.70–6.86 in Ma-3) results in robust potentiation of the channel over the pH range of 6.8 to 7.75, likely stabilizing the open state under these conditions. This effect is similar to PcTx1 at rASIC1a (14, 15); however, the PcTx1 outcome is dominated by stabilizing the desensitized state and current inhibition until the conditioning pH is higher than ∼7.6 where it shifts to potentiation. By contrast, Ma-3 has a dominant potentiating effect on hASIC1b over a broader pH range.

The nonselective ASIC inhibitor amiloride inhibits acid-induced pain in humans (16); however, the relative roles of homomeric and heteromeric ASIC1a, ASIC1b, and ASIC3 in peripheral nociceptors are unknown. Answering this question and progressing ASIC-targeting compounds to the clinic requires an understanding of their pharmacology at human ASICs. Despite the potential of mambalgins as ASIC1b-inhibiting peripheral analgesics in mice, we show that Ma-3 is not a potent inhibitor of heterologously expressed hASIC1b or 1b/3 heteromers, emphasizing the importance of preclinical human channel validation. Future studies with mambalgins and other ASIC modulators could assist in determining the ASIC subtype composition in human nociceptors at the functional protein level by comparing activity from primary cells with that of defined ASIC compositions. A single point mutation can convert PcTx1 from an inhibitor to potentiator of ASIC1a (15). Similarly, design of mambalgin analogs that potently inhibit hASIC1b could provide the required tools for human pain target validation and novel therapeutic leads.

## Materials and Methods

Ma-3 production and two-electrode voltage clamp procedures are described in *SI Appendix*, *Extended Methods*. Oocyte isolation was approved by the University of Queensland Anatomical Biosciences Animal Ethics Committee (QBI/AIBN/087/16/NHMRC/ARC). Data are mean ± SEM and *n* = 5 to 6.

## Supplementary Material

Supplementary File

## Data Availability

All study data are presented in the article.
