# Supplementary material for: Mambalgin-3 potentiates human acid-sensing ion channel 1b under mild to moderate acidosis: Implications as an analgesic lead
Source: Proc Natl Acad Sci U S A. 2021 Feb 19;118(8):e2021581118. doi: 10.1073/pnas.2021581118 (PMC7923528; doi:10.1073/pnas.2021581118)
Supplement: Supplementary File [file pnas.2021581118.sapp.pdf]

PNAS
www.pnas.org

Supporting information for:

Mambalgin-3 potentiates human acid-sensing ion channel 1b under mild to moderate acidosis: Implications as an analgesic lead

Ben Cristofori-Armstrong[a,1], Elena Budusan[a], and Lachlan D. Rash[a,1]

[a]School of Biomedical Sciences, Faculty of Medicine, The University of Queensland, St Lucia, QLD 4072, Australia

[1]Lachlan D. Rash and Ben Cristofori-Armstrong

Email: l.rash@uq.edu.a and b.cristoforiarmstrong@uq.edu.au

This PDF file includes:

Extended Methods
SI References

Extended Methods:

Mambalgin-3 production. Recombinant Ma-3 was expressed and purified in *E. coli* as previously described (1). The Ma-3 sequence (UniProt C0HJB0) is preceded by a tobacco etch virus (TEV) protease site including a vestigial non-native serine. Ma-3 specific changes to the published protocol include the use of a modified TN buffer (30 mM Tris, 300 mM NaCl, pH 8) and TEV buffer with 10 mM reduced and 1 mM oxidized glutathione.

Oocyte electrophysiology. Peptide activity was assessed using two-electrode voltage-clamp (TEVC) experiments performed on *Xenopus laevis* oocytes expressing rat (r) and human (h) ASIC channels. The ASIC clones used correspond to Uniprot identifiers P55926-1 (rASIC1a), P55926-3 (rASIC1b), P78348-2 (hASIC1a), P78348-3 (hASIC1b), and Q9UHC3-1 (hASIC3). These clones are comparable variants of rat and human ASIC1a and ASIC1b. cRNA was synthesized using an mMessage mMachine cRNA transcription kit (Ambion Inc., Austin, TX, USA). Healthy stage V-VI oocytes were injected with 0.5–5 ng cRNA for rASIC1a, rASIC1b, and hASIC1a or 2.5 ng cDNA for hASIC1b (cloned into the pMT3 expression vector) per oocyte for homomeric data. For heteromeric channels, hASIC1a, hASIC1b, and hASIC3 were cloned into the pUNIV backbone plasmid (2) and cRNA synthesized. We co-injected cRNA with ratios of 1:10 (hASIC1a:hASIC1b and hASIC1a:hASIC3), and 1:1 (hASIC1b:hASIC3) to account for the lower expression levels of hASIC1b and hASIC3 compared to hASIC1a. Total amounts of cRNA per oocyte were 2.5 ng for hASIC1a/1b, 1.5 ng for hASIC1a/3, 2 ng for hASIC1b/3. Previous work with ASIC1a and ASIC2a has shown that co-expression of these subtypes results in random assembly of trimers with a flexible stoichiometry of either 1:2 or 2:1 that cannot be discriminated by electrophysiology (3). Although we cannot exclude the presence of homomeric channels as a small population of trimers contributing to the observed current, the formation of heteromers is more likely. To ensure oocytes for heteromer data did not express homomeric hASIC1a or hASIC1b as the main population, the ratio of peak current magnitude evoked by stimulation with pH 6:pH 5 was tested for each oocyte before application of Ma-3. The measured current ratio for each heteromer combination robustly differed from that of homomeric hASIC1a (~0.8) or hASIC1b (~0.25) with equivalent amounts of cRNA injected, suggesting the major population to be heteromeric ASICs of the desired subtypes.

Oocytes were kept at 17°C in ND96 solution (96 mM NaCl, 2 mM KCl, 1.8 mM $CaCl_2$, 2 mM $MgCl_2$, 5 mM HEPES; pH 7.45) supplemented with 5 mM pyruvic acid, 50 µg/ml gentamicin, and 2.5% fetal horse serum. Experiments were performed at room temperature (21–22°C) 1–4 days after cRNA/cDNA injection in ND96 solution. HEPES was replaced by MES to buffer solutions at pH<6.8. Test solutions in the presence of Ma-3 contained 0.05% fatty acid free-bovine serum albumin (BSA) to prevent adsorption to plastic ware and tubing. The corresponding control conditions also contained 0.05% fatty acid free-BSA as a vehicle control. Oocytes were clamped at –60 mV (Axoclamp 900A; Molecular Devices, CA, USA) using microelectrodes filled with 3 M KCl solution (0.5–1.0 MΩ resistance). Data acquisition (sampled at 5 kHz and filtered at 0.01 Hz) was performed using pClamp 10 software (Molecular Devices, CA, USA). Concentration-response data were obtained with serial dilutions of Ma-3 applied at pH 7.45 for 55 s, and channels stimulated by a pH drop to 6.0 or 5.0 (as indicated for each data set) applied for 5 s without Ma-3. Activation curves were determined by conditioning oocytes at pH 7.45 for 55 s and applying a stimulus pH from 7.25 to 5.0 for 5 s. Steady-state desensitization (SSD) was determined by applying various conditioning solutions of pH values from 7.75 to 6.0 for 115 s prior to stimulation by a pH drop to 5.0 for 5 s (with the exception of Fig 2G where channels are stimulated with pH 6.0). For mechanism of action studies in the presence of Ma-3, the peptide is applied only in the conditioning solution for activation data (55 s application time) and SSD data (115 s application time). The exception is Fig 2E where Ma-3 is applied only in the stimulus solution, as indicated in the figure and text. Control data is obtained in the absence of Ma-3.

Changes in solution were induced using a gravity fed microperfusion system with a flow rate of ~1.5 mL/min to allow solution exchange (bath volume ~30 µL). Although efforts were made to

maximize flow rates in the experimental setup, solution exchange rates affect current kinetics due to the change in speed of ligand application (in this case protons). Ma-3 is a gating modifier of each ASIC tested, therefore it is likely the kinetics of currents are also modified to some degree. As such, the solution exchange rates are likely to have influenced the ASIC currents presented here. Due to this inherent experimental limitation of ligand-containing solution exchange around an oocyte affecting current kinetics, and the maximal speed of this solution being slower than the gating of single ion channels, we have focused our efforts on analyzing the peak and steady state currents rather than kinetics of traces. Further details are in the main text and below methods.

Animal work was carried out in strict accordance with the recommendations in the Australian code of practice for the care and use of animals for scientific purposes (8th Edition). Frog recovery surgery was performed under anesthesia (animals bathed in 1.3 mg/mL MS-222), and all efforts were made to minimize suffering. The minimum time between surgeries on the same animal was three months, and on the final surgery (maximum of six) frogs were euthanized by decapitation under MS-222 and ice anesthesia.

Data analyses. Data were analyzed in GraphPad Prism 8. The Hill equation (specifically the "sigmoidal dose-response (variable slope)" in Prism 8) was fit to the data with no constraints. The equation is Y = Bottom + (Top-Bottom)/(1+10^((LogEC50-X)*HillSlope)), where Bottom is the Y value at the bottom plateau, Top is the Y value at the top plateau, LogEC50 is the X value (the logarithm of [Ma-3] or pH units) response at the midpoint between Bottom and Top, and the HillSlope is the steepness of the curve (or the Hill coefficient; n_H). LogEC50 is the half-maximal response and refers to the LogEC50, LogIC50, or pH_{50} as appropriate for the data set. For the Ma-3 concentration-response data, after curve fitting to obtain the LogEC50/LogIC50, these values were converted to EC_{50}/IC_{50} and presented in molar (e.g. nM, μM) for better readability. pH_{50} values are kept in logarithmic units as per convention and the distribution of these data presented as 95% confidence intervals (95%CI).

Data were normalized before fitting. In Fig 1E and G, peak current (I) after Ma-3 conditioning were normalized to the peak current in control conditions (expressed as $I/I_{control\ peak}$), where peak current is defined as the maximal current magnitude observed during the 5 s of low pH stimulus. For some of the Ma-3 concentration-response data at hASIC1b and hASIC1b/3, although the data were fit with the Hill equation in the graph for visual representation, the EC_{50}/IC_{50} and Hill coefficient values were not reported due to the lack of reaching a plateau likely making these values erroneous. Currents remaining 5 s after low pH application are given as I_{5s}. In Fig 1H, values of I_{5s} when conditioned with Ma-3 are normalized to I_{5s} from control conditioning ($I_{5s\ Ma\text{-}3}/I_{5s\ control}$). In Fig 1I, I_{5s} is normalized to peak current of the same activation (I_{peak}), and this ratio is reported as I_{5s}/I_{peak}. Data in Fig 1I are not fit with the Hill equation, but a line connecting the data points between 10–1000 nM is shown. We find the degree of hASIC1b steady state current to be quite variable between oocytes for a given pH condition tested. This variability in the sustained current was also noted by the Askwith lab during their initial characterization of this channel (4). The importance of this conductance, and the reason for its variability when examined using oocyte electrophysiology is currently unknown. Nonetheless, our method of data analyses in Fig 1H and I examine the effect of Ma-3 on this steady-state conductance accounts for inter-oocyte variability. In Figure 2 current from each measurement were normalized to either the maximal control peak current ($I/I_{control\ max}$) or the maximal peak current observed in the presence of Ma-3 ($I/I_{Ma\text{-}3\ max}$). The Y value in the equation refers to each respective normalized current response as indicated in each figure. All data points on graphs are shown as mean ± SEM (standard error of the mean), the number of replicates (n) represent separate experimental oocytes. Data collection and analyses were not performed under blinding or randomization.

SI References:

1. N. J. Saez, B. Cristofori-Armstrong, R. Anangi, G. F. King, A Strategy for Production of Correctly Folded Disulfide-Rich Peptides in the Periplasm of E. coli. *Methods Mol Biol,* 1586, 155-180 (2017).
2. S. P. Venkatachalan *et al.*, Optimized expression vector for ion channel studies in Xenopus oocytes and mammalian cells using alfalfa mosaic virus. *Plufgers Arch*, 454(1), 155-163 (2007).
3. T. Bartoi, K. Augustinowski, G. Polleichtner, S. Gründer, M. H. Ulbrich, Acid-sensing ion channel (ASIC) 1a/2a heteromers have a flexible 2:1/1:2 stoichiometry. *Proc Natl Acad Sci U S A,* 111 (22), 8281-8286 (2014).
4. E. N. Hoagland, T. W. Sherwood, K. G. Lee, C. J. Walker, C. C. Askwith, Identification of a calcium permeable human acid-sensing ion channel 1 transcript variant. *J Biol Chem,* 285, 41852-41862 (2010).
